# Supplemental *Bacillus subtilis* DSM 32315 manipulates intestinal structure and microbial composition in broiler chickens

**DOI:** 10.1038/s41598-018-33762-8

**Published:** 2018-10-18

**Authors:** Youbiao Ma, Weiwei Wang, Haijun Zhang, Jing Wang, Wenming Zhang, Jun Gao, Shugeng Wu, Guanghai Qi

**Affiliations:** 1 Key Laboratory of Feed Biotechnology of Ministry of Agriculture, Feed Research Institute, Chinese Academy of Agricultural Sciences, Beijing, 100081 China; 2 Evonik Degussa (China) Co. Ltd., Beijing, 100026 China

## Abstract

Knowledge about the modulation of gut microbiota improves our understanding of the underlying mechanism by which probiotic treatment benefits the chickens. This study examined the effects of *Bacillus subtilis* DSM 32315 on intestinal structure and microbial composition in broilers. Broiler chicks were fed basal diets without or with *B*. *subtilis* supplementation (1.0 × 10^9^ spores/kg of diet). Supplemental *B*. *subtilis* increased average body weight and average daily gain, as well as elevated villus height and villus height to crypt depth ratio of ileum in broilers. Multi-dimension analysis showed a certain degree of separation between the cecal microbiota from treatment and control groups. Increased *Firmicutes* abundance and reduced *Bacteroidetes* abundance in cecum were observed responded to *B*. *subtilis* addition, which also increased the abundances of *Christensenellaceae* and *Caulobacteraceae*, and simultaneously decreased the abundances of potentially harmful bacteria such as *Vampirovibrio*, *Escherichia/Shigella* and *Parabacteroides*. Network analysis signified that *B*. *subtilis* addition improved the interaction pattern within cecal microbiota of broilers, however, it exerted little influence on the metabolic pathways of cecal microbiota by comparison of the functional prediction of metagenomes. In conclusion, supplemental *B*. *subtilis* DSM 32315 improved growth performance and intestinal structure of broilers, which could be at least partially responsible by the manipulation of cecal microbial composition.

## Introduction

The current tendency in animal production is towards a reduction or banning of the use of antibiotics, and an increase in application of non-antibiotic approaches that can provide similar benefits, due to that widespread use of antibiotics has led to the emergence of resistant bacteria and drug residues in animal products. In this context, researchers have been compelled to find alternates to ongoing therapeutic regimes, mainly dependent over antibiotics. There was an interest to characterize the probiotics as a kind of viable alternatives to promote growth and health status of poultry by multiple ways^1^. Despite a large amount of microorganisms served as probiotics in poultry production, the form of supplemental probiotics through the hostile environment such as low pH value and high concentration of bile salt within gastrointestinal tract are a severe challenge for their survival^2^. As a result, spore-forming bacteria such as *Bacillus subtilis* are gaining interest in animal health related functional additive research, due to their high tolerance and survivability under hostile environment in gastrointestinal tract^3^. An obvious advantage of *B*. *subtilis* applied in feed is the stability and extended shelf life without losing viability. Supplemental *B*. *subtilis* has earned many benefit claims, including the immune-modulation, promotion of nutrients digestibility, along with improvements of intestinal health and growth performance in animals^4,5^. However, many properties of probiotic bacteria vary as a function of strain^6^. With respect to *B*. *subtilis*, its probiotic effects are highly strain-specific and the mechanistic details remain largely elusive^7^. It was reported that the effects of *B*. *subtilis* supplementation on growth performance and intestinal physiology in broilers were markedly strain-dependent^8,9^, providing a need for the continuous studies on the various strains of *B*. *subtilis* to understand their respective properties in animals. *B*. *subtilis* DSM 32315, a unique probiotic strain, was derived from a multi-parameter selection process, in which more than 500 environmentally sampled *Bacillus* strains were screened. This strain exhibited several distinct properties that are vital for its beneficial effects in gastrointestinal tract^10^. The preliminary trial has shown an improvement of growth performance in broilers associated with *B*. *subtilis* DSM 32315 addition^11^. However, far less is known about the effects of this strain on gut health in chickens.

Gut microbiota, an important inhabitant of host, has profound impacts on the bioavailability of dietary components, playing prominent roles in host nutritional and physiological processes^12^. A full picture of gut microbiota may be crucial for the dietary intervention to promote growth performance and health status of host^12^. It was thought that the improvements growth performance and intestinal functions of animals by probiotics could be established by the ability of balancing gut microflora which helped to attenuate intestinal inflammation and recover intestinal mucosa from injury^13,14^. Previous studies in chickens indicated a change in bacterial enumeration in gut after *B*. *subtilis* supplementation^5,15^, however, a comprehensive understanding is lacking about the shift of the composition and functionality of gut microbiota with *B*. *subtilis* addition. In keeping with this, the main objective of this study was to investigate the effects of supplemental *B*. *subtilis* DSM 32315 on intestinal microbial composition, which may subsequently benefit intestinal structure and growth performance of broilers.

## Results

### Growth performance

Supplemental *B*. *subtilis* DSM 32315 significantly increased (*P* < 0.05) average body weight (ABW) of broilers at 28 and 42 d of age (Table 1), along with average daily gain (ADG) of birds during the grower period (1−28 d) and overall period (1−42 d). Besides, average daily feed intake (ADFI) during the overall period was significantly higher (*P* < 0.05) in birds receiving *B*. *subtilis* supplementation. However, no significant difference (*P* > 0.05) in feed conversion ratio (FCR) of birds was observed between the two groups during the grower or overall period.

**Table 1:** Effects of *Bacillus subtilis* DSM 32315 addition on growth performance in broiler chickens (*n* = 10). ABW, average body weight; ADG, average daily gain; ADFI, average daily feed intake; FCR, feed conversion ratio. ^a,b^Different letters within rows indicate differences between treatment groups (*P* < 0.05).

		Control	*B*. *subtilis*	*P-*value
28 d	ABW (g)	1430 ± 38^b^	1469 ± 38^a^	0.033
42 d	ABW (g)	2670 ± 97^b^	2774 ± 89^a^	0.022
Grower 1−28 d	ADG (g)	49.2 ± 1.3^b^	50.6 ± 1.4^a^	0.028
	ADFI (g)	67.5 ± 1.9	68.9 ± 2.1	0.135
	FCR	1.37 ± 0.04	1.36 ± 0.02	0.381
Overall 1−42 d	ADG (g)	60.4 ± 2.5^b^	62.7 ± 2.3^a^	0.041
	ADFI (g)	89.9 ± 2.7^b^	93.1 ± 2.8^a^	0.017
	FCR	1.49 ± 0.04	1.49 ± 0.02	0.740

### Intestinal histomorphology

**Figure 1:**
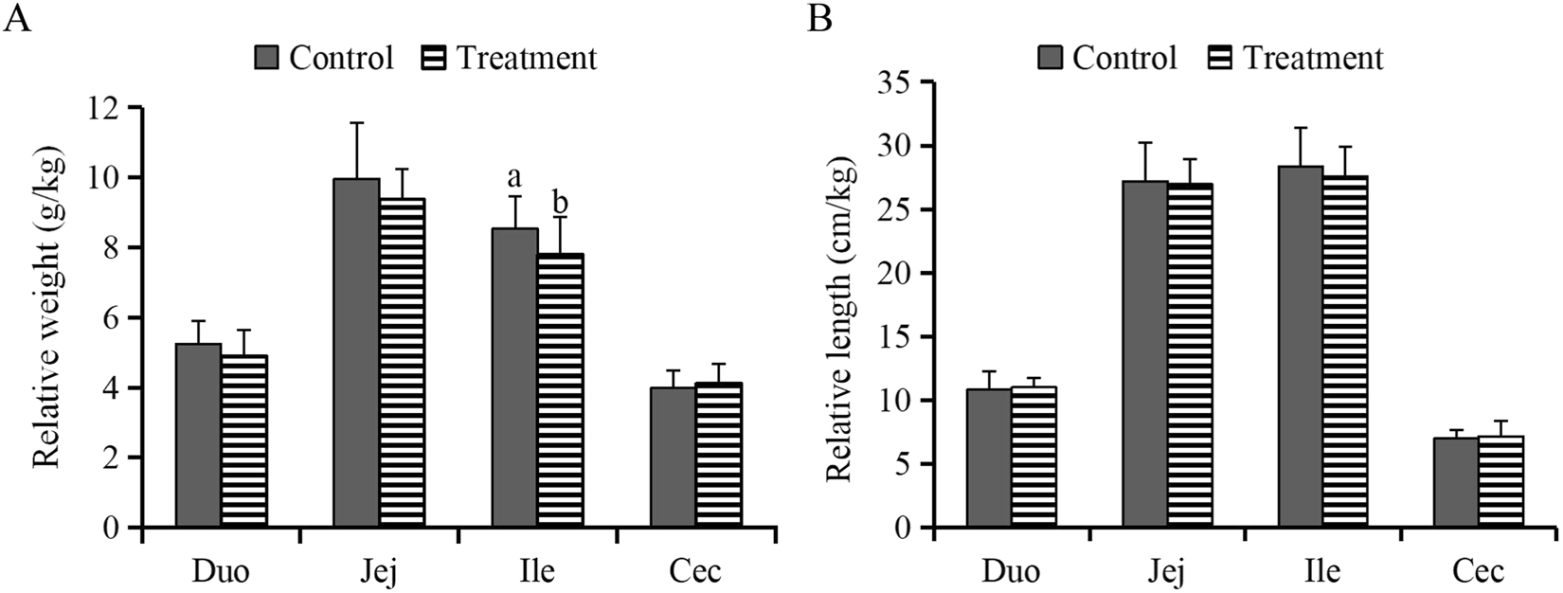
Effects of *Bacillus subtilis* DSM 32315 addition on the relative weight (**A**) and relative length (**B**) of various intestinal segments in broiler chickens (*n* = 10). Relative weight of intestines was expressed as the ratio of intestinal weight (g) to body weight (kg). Relative length of intestines was expressed as the ratio of intestinal length (cm) to body weight (kg). Duo, duodenum; Jej, Jejunum; Ile, ileum; Cec, cecum. ^a,b^Mean values with unlike letters indicate differences between treatment groups (*P* <0.05).

Supplemental *B*. *subtilis* DSM 32315 significantly decreased (*P* < 0.05) the relative weight of ileum of broilers (Fig. 1A), but it had no significant influence (*P* > 0.05) on the relative weight of duodenum, jejunum, and cecum, or the relative length of these intestinal segments (Fig. 1A,B). The villus height (VH) and VH-to-crypt depth (CD) ratio (VCR) of ileum rather than of duodenum and jejunum were significantly higher (*P* < 0.05) in treatment group relative to control (Table 2).

**Table 2:** Effects of *Bacillus subtilis* DSM 32315 addition on intestinal morphology in broiler chickens (*n* = 10). VH, villus height; CD, crypt depth; VCR, villus height to crypt depth ratio. ^a,b^Different letters within rows indicate differences between treatment groups (*P* < 0.05).

		Control	*B*. *subtilis*	*P-*value
Duodenum	VH (µm)	895.1 ± 211.8	894.3 ± 138.3	0.992
	CD (µm)	174.7 ± 26.1	195.6 ± 26.3	0.092
	VCR	5.12 ± 0.95	4.63 ± 0.88	0.248
Jejunum	VH (µm)	688.0 ± 118.9	730.6 ± 102.3	0.402
	CD (µm)	142.2 ± 24.1	163.7 ± 40.9	0.169
	VCR	4.92 ± 0.98	4.61 ± 0.84	0.464
Ileum	VH (µm)	421.9 ± 58.3^b^	551.6 ± 110.9^a^	0.004
	CD (µm)	101.2 ± 10.0	109.0 ± 24.9	0.365
	VCR	4.18 ± 0.47^b^	5.12 ± 0.62^a^	0.001

### Relative mRNA expression of ileal genes

**Figure 2:**
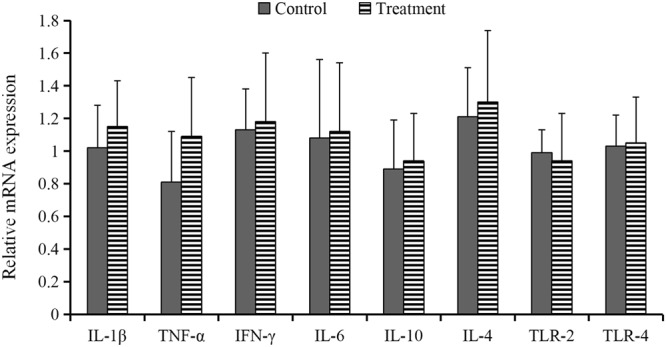
Effects of *Bacillus subtilis* DSM 32315 addition on intestinal lesion scoring (**A**) and relative mRNA expression of inflammation-related genes of ileum (**B**) in broiler chickens (*n* = 10).

As indicated in Fig. 2, there were no significant changes (*P* > 0.05) in the expression of tumor necrosis factor (TNF)-α, interleukin (IL)-1β, IL-6, IL-10, IL-4, interferon (IFN)-γ, toll-like receptor (TLR)-4 and TLR-2 in ileum responded to *B*. *subtilis* DSM 32315 addition.

### Diversity of cecal microbiota

**Figure 3:**
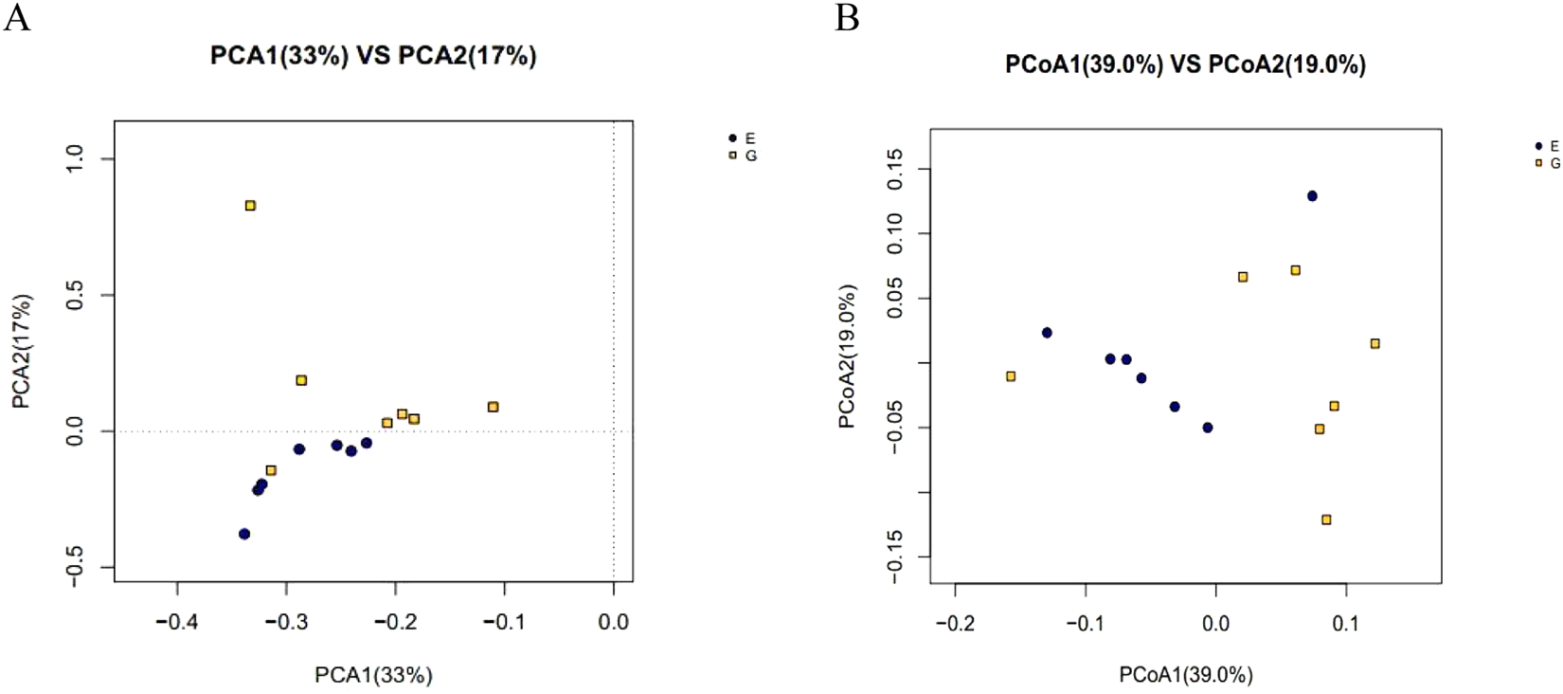
Beta-diversity analysis of microbial communities by using principal component analysis (PCA) based on Operational Taxonomic Units (OTUs) and principal co-ordinates analysis (PCoA) based on weighted UniFrac distances (*n* = 7). Abscissa represents the first principal component, ordinate represents the second principal component, and the percentage represents the contribution of the principal component to the sample difference. E, control group; G, treatment group.

Coverage indice, used to evaluate the percentage of total bacterial Operational Taxonomic Units (OUTs) represented in a sample, were over 99.7% (data not shown), indicating the current 16S rDNA results from each library could represent the complete microbial communities of cecum. However, the α-diversity including Shannon, Simpson, ACE, and Chao1 indexes as well as richness diversity of cecal microbiota were not significantly different (*P* > 0.05) between treatment and control groups (Supplementary Fig. S1). To estimate the similarity (β-diversity) of microbial community structure between groups, principal component analysis (PCA) based on OUT profile and principal coordinate analysis (PCoA) based on weighted UniFrac distance were performed. As presented in Fig. 3A, PCA plot defined groups where the samples from control and treatment groups occupied distinct positions. The first axis of the PCA explained 33% of the variation in microbial diversity while the second axis explained 17% of it. PCoA plot also showed a trend of separation of microbial communities between the two groups (Fig. 3B).

### Composition of cecal microbiota

**Figure 4:**
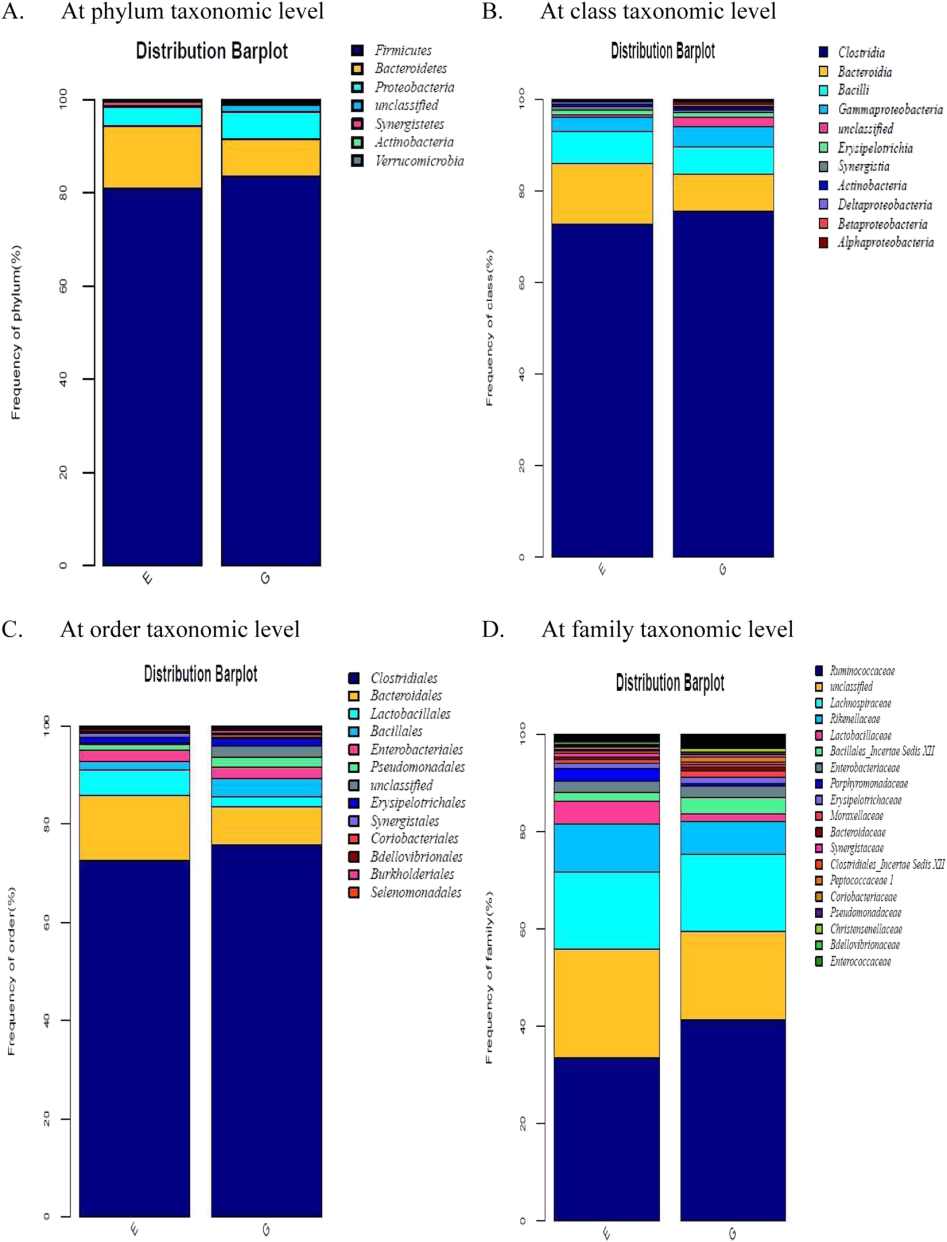
Effects of *Bacillus subtilis* DSM 32315 addition on cecal microbial composition at different taxonomic levels in broiler chickens (*n* = 7). E, control group; G, treatment group.

The relative abundance of OTUs of cecal microbiota was analysed at different ranking levels from phylum to genus. The dominant phyla across the two groups were *Firmicutes*, *Bacteroidetes*, and *Proteobacteria*, together contributing greater than 95% of the whole phyla (Fig. 4A). Birds supplemented with *B*. *subtilis* had a higher abundance of *Firmicutes* and a lower abundance of *Bacteroidetes*. Class level microbiota analysis showed that the cecal microbiota was dominated by *Clostridia* followed by *Bacteroidia* and *Bacilli*, which collectively occupied more than 90% of the total sequences (Fig. 4B). Within phylum *Firmicutes*, the abundance of *Clostridia* was higher while *Bacilli* abundance was lower in treatment group relative to control, which was concomitant with a depletion of *Bacteroidetes* in treatment group. At order taxonomic level (Fig. 4C), *Clostridiales* and *Bacteroidales* accounted for the largest proportion of the community, which were respectively increased and decreased by *B*. *subtilis* addition. Within *Clostridiales*, the majority belonged to the *Ruminococcaceae* and *Lachnospiraceae* families (Fig. 4D). An increase of *Ruminococcaceae* abundance responded to *B*. *subtilis* treatment occurred.

**Figure 5:**
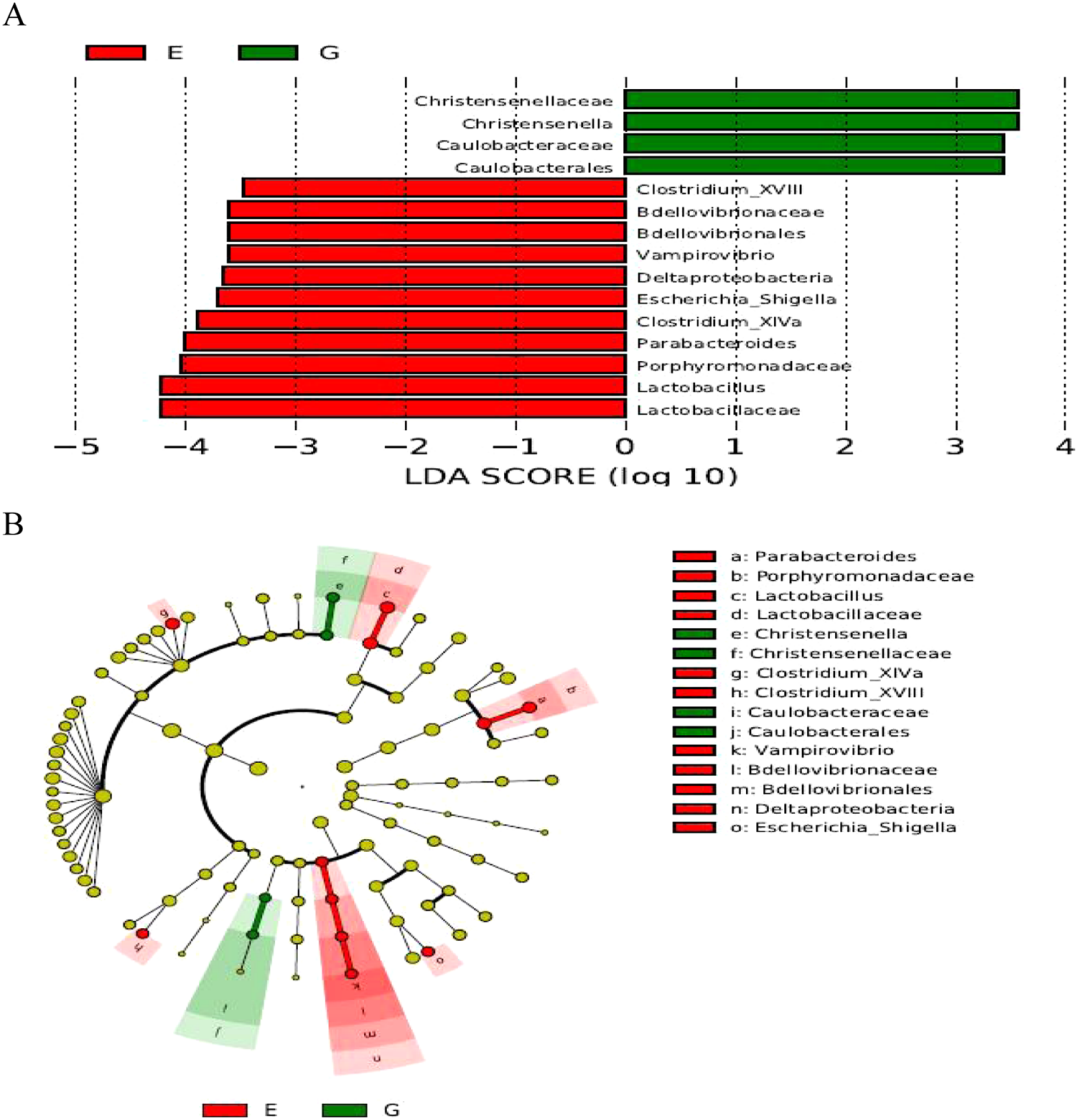
Linear discriminant analysis (LDA) combined effect size measurements (LEfSe) analysis of cecal microbiota in broiler chickens (*n* = 7). (**A**) Species with significant difference that have an LDA score greater than the estimated value (the default score is 2.0). The length of the histogram represents the LDA score. (**B**) The cladogram diagram shows the microbial species with significant differences between the two groups. The species classification at the level of phylum, class, order, family, and genus exhibit from the inside to the outside. Red and green nodes in the phylogenetic tree represent microbial species that play an important role in control and treatment groups, respectively. Yellow nodes represent species with no significant difference. E, control group; G, treatment group.

We further applied linear discriminant analysis (LDA) combined effect size measurements (LEfSe) to explore the relative richness (*P* < 0.05, LDA > 2.0) of bacterial members in control or treatment groups. As presented in Fig. 5, *Christensenella* (*Christensenellaceae*) and *Caulobacterales* (*Caulobacteraceae*) were enriched in cecum of treatment group, whereas control microbiota was differentially enriched with *Clostridium* XVIII, *Bdellovibrionales* (*Bdellovibrionaceae*), *Vampirovibrio*, *Deltaproteobacteria*, *Escherichia/Shigella*, *Clostridium* XIVa, *Parabacteroides*, *Porphyromonadaceae* and *Lactobacillaceae* (*Lactobacillus*).

### Network pattern of cecal microbiota

**Figure 6:**
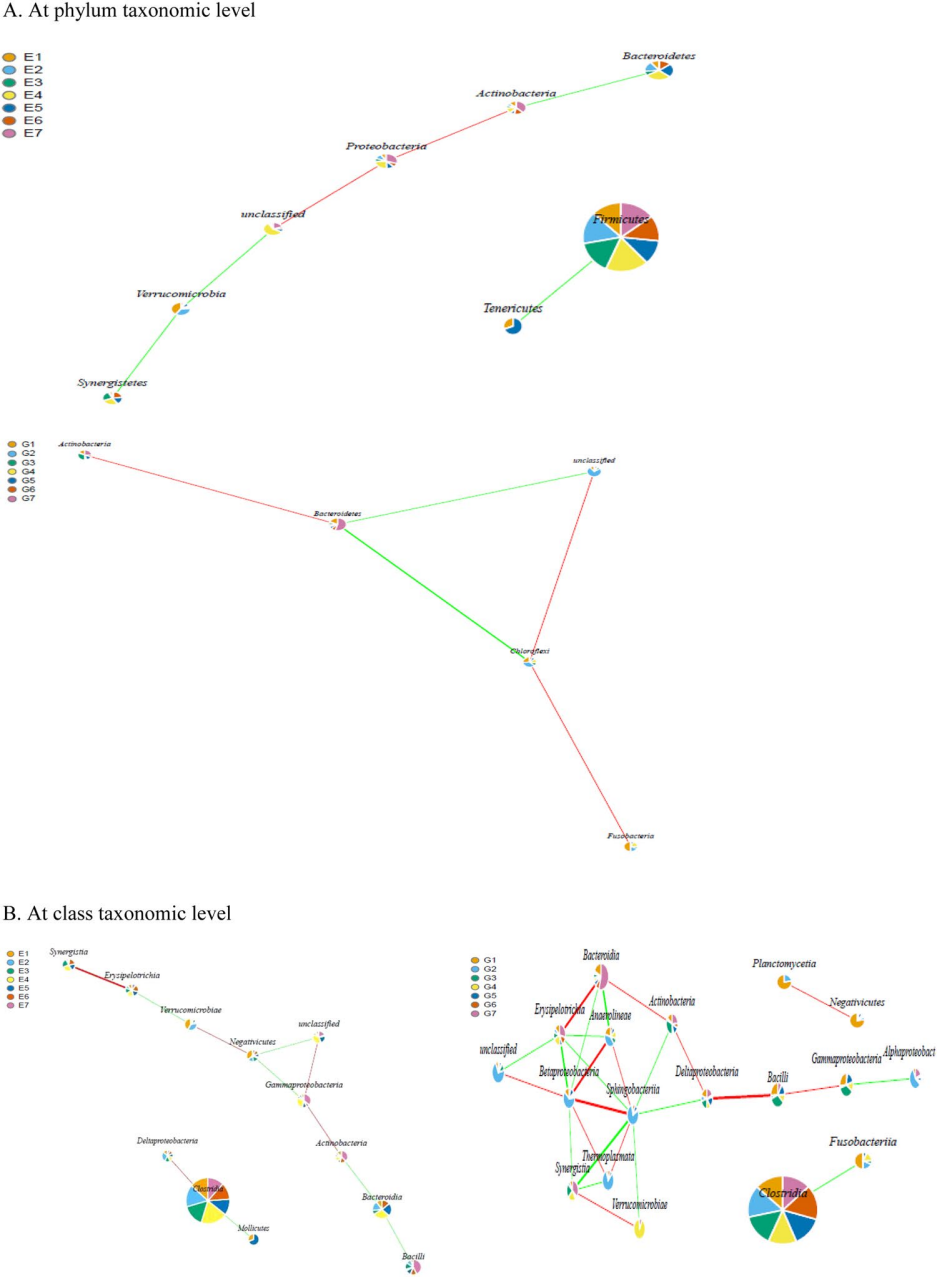
The co-network pattern of cecal microbiota at phylum (**A**) and class (**B**) levels in broiler chickens (*n* = 7). The significance of correlation was set as *P* <0.05 and expressed by the dotted line, while the high significance of correlation was set as *P* <0.01 and expressed by the full line. The red and green lines among nodes stand for the positive and negative correlations, respectively. The weight of the lines correspond to the correlation coefficients, whose values were higher and lower than 0.8 were represented by the thick and thin lines, respectively. The diameter of the nodes was proportional to the relative abundance. Taxa in brackets are based on annotations suggested by the Greengenes database. E, control group; G, treatment group.

We employed correlation network analysis to identify interactions between gut microbiota that changed with administration of *B*. *subtilis* DSM 32315. As shown in Fig. 6A, there was a negative correlation between *Firmicutes* and *Tenericutes*, as well as between *Bacteroidetes* and *Actinobacteria* in control microbiota. However, a destruction to this network was found in treatment group, in which *Bacteroidetes* was positively and negatively correlated with *Actinobacteria* and *Chloroflexi*, respectively, while no correlation was detected between *Firmicutes* and other members. Besides, a more complicate microbial interaction pattern that evidenced by the presence of more core nodes (such as *Sphingobacteria*, *Betaproteobacteria* and *Erysipelotrichia* that had the highest number of connections with the rest of bacterial members) was found in response to *B*. *subtilis* DSM 32315 addition (Fig. 6B). For control group, *Clostridia* was positively and negatively correlated with *Deltaproteobacteria* and *Mollicutes*, respectively, while *Bacteroidia* was negatively correlated with *Bacilli* and *Actinobacteria*. In treatment group, however, there was a negative correlation between *Clostridia* and *Fusobacteriia*, as well as a highly positive correlation between *Bacilli* and *Deltaproteobacteria*. Remarkably, *Bacteroidia* was positively correlated with both *Actinobacteria* and *Erysipelotrichia*. Moreover, the classes *Sphingobacteria*, *Betaproteobacteria* and *Erysipelotrichia* had the highest number of connections with the rest of the community in treatment group. Network analysis at both order and family taxonomic levels also revealed more complex co-occurrence relationships within cecal microbiota of treatment group in comparison with control, as exhibited by a higher number of edges in the network from treatment group than that from control group (Supplementary Fig. S2).

### Functional prediction of cecal microbiota

**Figure 7:**
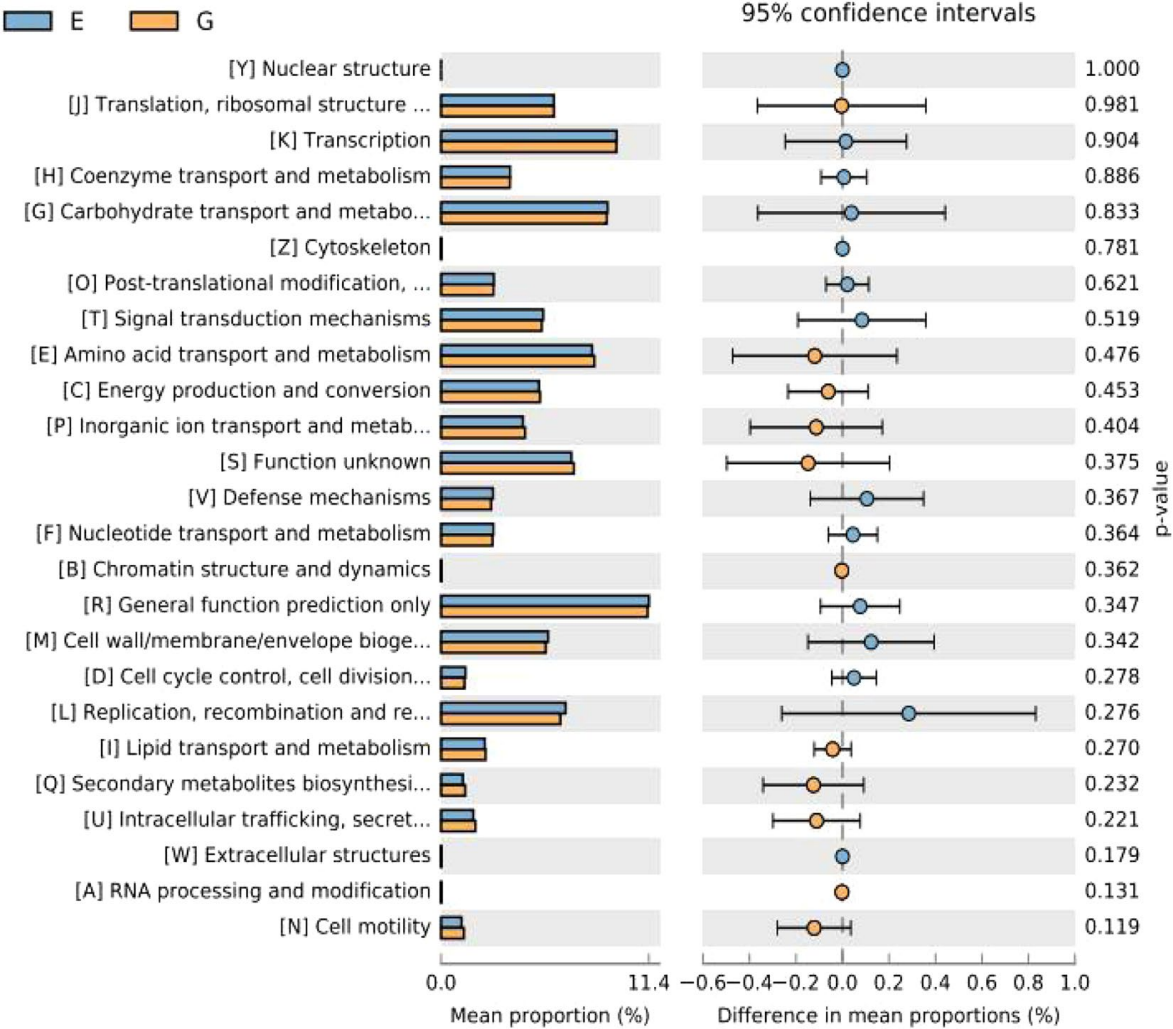
Functional comparison of cecal metagenomic sequence based on Clusters of Orthologous Groups (COG) categories in broiler chickens (*n* = 7). The differences between the levels of the predicted functions were tested using a two-sided Welch’s t test. E, control group; G, treatment group.

**Figure 8:**
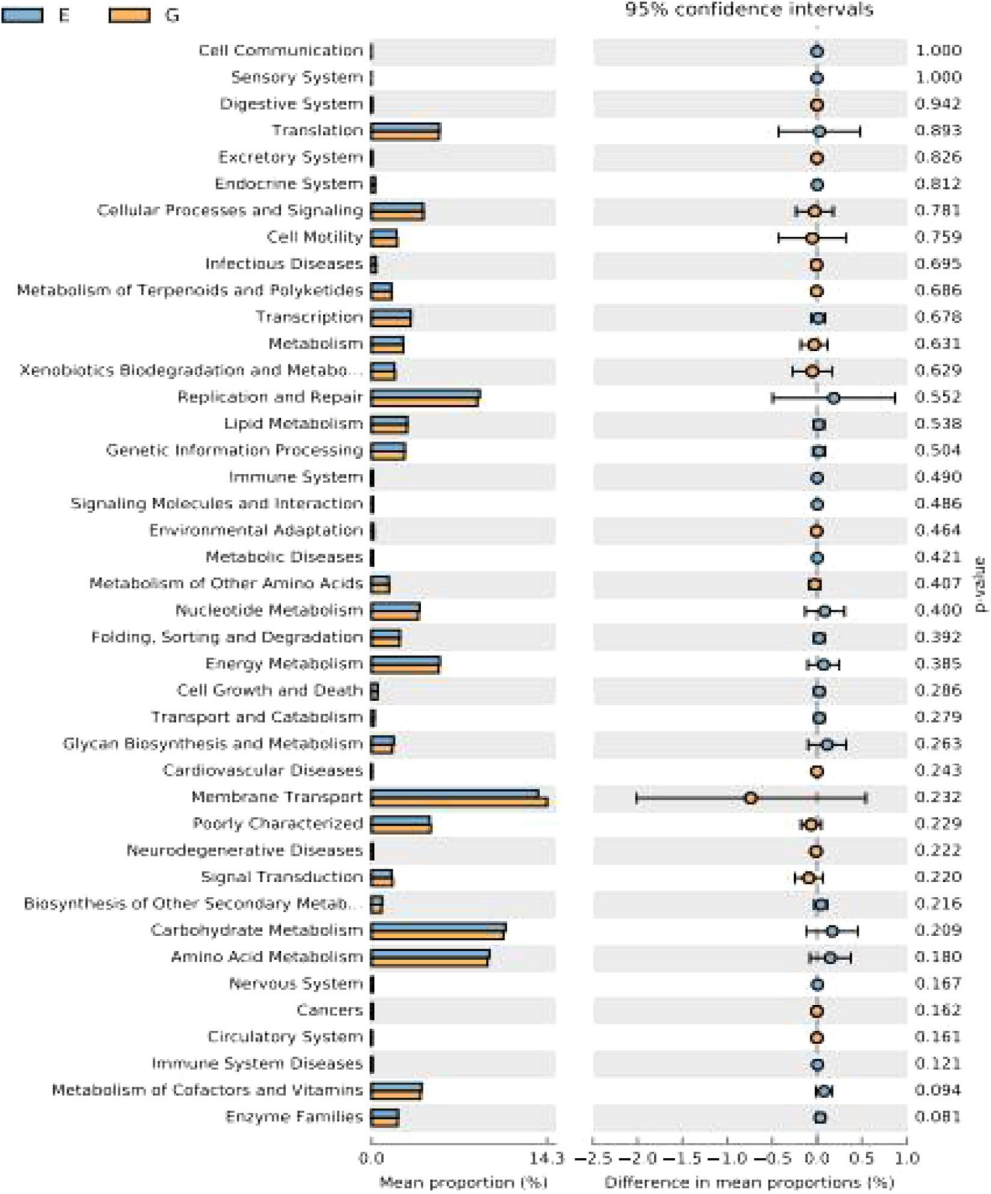
Functional comparison of cecal metagenomic sequence based on Kyoto Encyclopedia of Genes and Genome (KEGG) orthologs in broiler chickens (*n* = 7). The differences between the levels of the predicted functions were tested using a two-sided Welch’s t test. E, control group; G, treatment group.

The changes in the presumptive functions of cecal microbiota were examined by predicting the metagenomes using Phylogenetic Investigation of Communities by Reconstruction of Unobserved States (PICRUSt). Before the examination, the functional accumulation curve was employed to evaluate the sufficiency of sampling number and the richness of functional components, as it can reflect the rate of emergence of new COG (Clusters of Orthologous Groups) categories or KEGG (Kyoto Encyclopedia of Genes and Genome) orthologs responded to the continuous sampling. As exhibited in Supplementary Fig. S3, the functional accumulation curve rise rapidly in the case of small sample and become smooth while the sampling number growing, indicating a plenitude of sampling in the present study. Metagenomic prediction based on COG categories revealed that the functional pathways enriched within cecal microbiota of broilers were general function prediction only, transcription, carbohydrate transport and metabolism, amino acid transport and metabolism, replication, recombination and repair, cell wall/membrane/envelope biogenesis (Supplementary Fig. S4). In terms of the prediction based on KEGG orthologs, the abundant functional annotations of cecal microbiota were those corresponding to membrane transport, carbohydrate metabolism, amino acid metabolism, replication and repair, translation, energy metabolism (Supplementary Fig. S5). However, there was little difference in the predicted pathways between the microbiota from treatment and control groups based on COG categories or KEGG orthologs through Welch’s t-test (Figs 7 and 8) and heatmap analysis (Supplementary Figs S6 and S7).

## Discussion

Growing evidences indicated that administration of *B*. *subtilis* into diets improved the growth performance of animals^4,5,8^. Contradictorily, some other studies found no relation of *B*. *subtilis* to growth performance of chickens^16,17^. It could be speculated that the effects of *B*. *subtilis* treatment are uncertain depending largely on various factors such as the bacterial strains^4^, since there was a distinction between the effects of different strains of *B*. *subtilis* on chicken performance^4^. Herein, we found an improvement of growth performance, as characterized by the increased ADG of broilers in the presence of *B*. *subtilis* DSM 32315, whose efficacy may derive from a modulation on the intestinal environment^4,18^. To verify this, we next investigated the shift of intestinal structure and microbial composition of birds with *B*. *subtilis* DSM 32315 addition.

Improvement of intestinal morphology including elevated VH and VCR suggest an increased absorptive surface area and expression of brush border enzymes that capable of greater digestion and absorption^19^, conducing to the improved growth performance in animals. In concert with a previous study^5^, we found increases in ileal VH and VCR responded to *B*. *subtilis* DSM 32315 addition, which might be partially responsible for the elevated ADG and ABW of broilers. The relative weight and relative length of intestinal segments are important estimators of gut histology. Herein, supplemental *B*. *subtilis* DSM 32315 decreased the relative weight of ileum in broilers, which was similar to the study of Reis *et al*.^20^ who reported an improved growth performance concomitant with a lessened relative weight of duodenum of broilers after *B*. *subtilis* (DSM 17299) addition. Increased weight of intestine could be an adaptive response to an increased need for digestion and absorption^21^. It was confirmed that supplementing some functional additives such as enzymes to diets could promote digestion and absorption, but which potentially attenuate the secretory function of intestine, leading to a reduction of the organ weight in animals^22^. *B*. *subtilis* was proposed to secrete a wide range of digestive enzymes within intestinal tract aid in digestion and absorption^23^. Thus, the present reduction of the relative weight of ileum may result from an enhanced absorption capacity, as supported by the improvement of ileal morphological structure with *B*. *subtilis* DSM 32315 addition.

Cytokines such as IL-1β and TNF-α are small immunoregulatory peptides that produced mainly by the activated macrophages or T lymphocytes through the activation of TLRs signaling pathways, aiding cell-to-cell communication during cellular immune responses^24^. *B*. *subtilis* was documented to trigger production of several kinds of cytokines, acting as the response of mononuclear cells to the stimulus contained in probiotic bacteria^23^. However, we found that *B*. *subtilis* DSM 32315 exerted little effect on the expression of ileal cytokines and TLRs, which was not in accordance with a previous study regarding the immunoregulation of *B*. *subtilis* B10 addition in broilers^25^. This discrepancy may be attributed to differences in the bacterial strains.

Gut microbiota conduce to the maintenance of normal physiological structure and function of intestinal tract, providing a variety of benefits for their host^12–14^. It is generally recognized that the improved growth performance and intestinal structure by probiotics are largely ascribed to a healthy modulation of gut microbiota^13,26^. In chickens, it was observed a change in bacterial enumeration in gut responded to *B*. *subtilis* addition^5,15^, however, to what extent it can influence the composition and functionality of gut microbiota is poorly understood. In the current study, though *B*. *subtilis* DSM 32315 addition failed to modify the α-diversity of cecal microbiota of broilers, it conferred several changes to the microbial composition, which were evidenced by a higher abundance of *Firmicutes* and a lower abundance of *Bacteroidetes* in treatment group, accompanied by the increased ABW and ADG of broilers. *Firmicutes* plays influential roles in polysaccharide decomposition and energy utilization in gut because of genes coding for the secretion of glycan-degrading enzymes^27^. Moreover, growth performance of animals was positively correlated with the abundance of *Firmicutes* especially the ratio of it to *Bacteroidetes* abundance in gut^13,28^. Hence, the elevation in *Firmicutes* to *Bacteroidetes* ratio in treatment group, reflected by the relevant increase in *Firmicutes* and reduction of *Bacteroidetes*, could at least partially explain the observed increases in ABW and ADG of broilers fed with *B*. *subtilis* DSM 32315. *Clostridia* comprises mostly non-pathogenic commensal bacteria including the members that capable of degrading complex glycans and proteins, connecting with the improved growth performance of chickens^29,30^. *Ruminococcaceae*, one of the most abundant family from the order *Clostridiales* in gut, has a high copy of genes equipped to degrade a serious of glycans such as cellulose^31^. Therefore, the increased abundances of *Clostridia* and *Ruminococcaceae* were likely associated with the increased ADG and ABW due to *B*. *subtilis* 32315 addition. LEfSe analysis identified more representative species as biomarkers to distinguish the microbiota of these two groups, including *Christensenellaceae* and *Caulobacteraceae*, along with *Clostridium* XVIII, *Vampirovibrio*, *Deltaproteobacteria*, *Escherichia/Shigella*, *Parabacteroides*, *Porphyromonadaceae* and *Lactobacillus*. *Christensenellaceae* was proposed to be associated with feed intake in animals^32^, whose reduction in gut was observed in reaction to gut inflammation^33^. *Caulobacteraceae* was characterized as the microorganism responsible for decomposing cellulose in ecosystems^34^. *Clostridium* XVIII was proved to be increased in the patients with bowel movement disorder^35^, highlighting a negative role of this bacterium in the regulation of gut health. The genus *Vampirovibrio* and class *Deltaproteobacteria* were highly enriched in gut of infected chickens, and associated with intestinal inflammation and injury^36,37^. *Escherichia*/*Shigella*, as opportunistic pathogenic bacteria, were described to destroy intestinal structure and exert pro-inflammatory activities through multiple ways such as the production of virulence factors^38^, resulting in an increased risk to the infection and diarrhea of host^39^. *Parabacteroides* also represents an opportunistic pathogen in gut, due to its frequent involvement in infectious diseases together with its ability to develop resistance to antimicrobial drugs^40^. Besides, *Parabacteroides* was considered to be linked with the reduction of body weight of host animals^41^. The increased *Porphyromonadaceae* was identified as a response to gut inflammation and tumorigenesis^42^, leading to an elevated risk of the occurrence of several intestinal diseases^43^. Taken together, the increased abundances of *Christensenellaceae* and *Caulobacteraceae*, along with the decreased abundances of *Clostridium* XVIII, *Deltaproteobacteria*, *Escherichia/Shigella*, *Parabacteroides*, *Porphyromonadaceae* in gut of treatment group could contribute to the improved growth performance and intestinal structure of broilers fed with *B*. *subtilis* DSM 32315. Strikingly, there was a reduction of *Lactobacillus* abundance of broilers with *B*. *subtilis* DSM 32315 addition. *Lactobacillus* is generally used as a kind of probiotics in animal production, as some strains of it can exert several benefits for host^1,2^. However, the negative correlation was also noted between certain strains of *Lactobacillus* and chicken growth performance^13^. There might be a positive relationship between *Lactobacillus* abundance and pathogen-induced gut dysfunction^44^, based on an enrichment of *Lactobacillus* in gut after enteric infection by pathogenic bacteria such as *Salmonella*^44,45^. The reasons for the reduced abundance of *Lactobacillus* in treatment group are presumably involved with the complex microbial-host milieu. One possibility is that the reduced *Lactobacillus* members in this study are functionally different from the strains used as probiotics. Alternatively, the reduction of *Lactobacillus* may be a feedback of the lessened potential pathogens and alleviated gut inflammation after *B*. *subtilis* DSM 32315 addition^46^.

Dynamic balance in microbial ecosystem depends on the interactions between bacteria and host environment as well as among bacterial members^47^. To our knowledge, no published study was available regarding the alteration of microbe-microbe interactions of gut microbiota induced by dietary intervention in broilers. Network analysis of taxon co-occurrence patterns provides new insights into the internal structure of complex microbial communities^48^. The positive correlations indicate cooperative interactions or the presence of common biological functions between taxa, while negative correlations could be indicative of competitive interactions between taxa^48^. A previous study confirmed an improved interaction pattern of gut microbiota in pigs fed with yeast probiotic^49^. In the present study, a diet-dependent co-occurrence network of cecal microbiota in broilers was observed. For example, there was a negative correlation between *Tenericutes* and *Firmicutes* in control group, while this microbial linkage observed in treatment group was absent. Besides, *B*. *subtilis* DSM 32315 addition increased relative degree of connectance of *Bacteroidetes* within the microbiota, and reversed the negative correlation between *Actinobacteria* and *Bacteroidia*, as well as promoted the positive correlation of *Bacilli* and *Actinobacteria* with some other bacteria, suggesting that *B*. *subtilis* DSM 32315 addition could fortify the interaction between *Actinobacteria* and other members, who can benefit from the metabolic activity of each other, namely mutualistic relationship. Such communities can thereby effectively utilize nutrients within intestinal tract and suppress the colonization by opportunistic bacteria^50^. In addition, *Actinobacteria* was regarded as “keystone taxa” that exert essential roles in modulating the functionality of gut microbiota and as a key source of novel bacteriocines and other metabolites, which were helpful for the maintenance of overall microbial structure, along with host growth and intestinal health^51,52^. Thereby, the potential inhibition of *Actinobacteria* by *Bacteroidetes* (as the second abundant phylum) in control group might be adverse to the performance and gut health of broilers, but which could be attenuated by *B*. *subtilis* DSM 32315 addition. With regard to the network analysis at order and family levels, higher degree of connectance of bacterial members within the community also occurred in treatment group, which agreed with the analysis results at phylum and class levels. To sum up, supplemental *B*. *subtilis* DSM 32315 resulted in a more intricate interaction pattern of cecal microbiota, which might be translated into improvements of growth performance and gut health of broilers^49^.

Commensal bacteria might increase or decrease certain specific metabolic pathways to react to the change in host intestinal environment that can be induced by dietary intervention in broilers^53^. In order to infer the alterations of metabolic pathways of cecal microbiota in response to *B*. *subtilis* addition, the PICRUSt analysis was employed in the present study. Unexpectedly, broilers shared a core set of predicted metagenomes of cecal microbiota regardless of *B*. *subtilis* addition, suggesting a stability of the functional capabilities of cecal microbiota in broilers. The metagenomic prediction based on COG categories revealed that cecal microbiota of broiler was enriched with the functional pathways associated with general function prediction only, transcription, carbohydrate transport and metabolism. In terms of the prediction based on KEGG orthologs, the abundant functional annotations of cecal microbiota were those corresponding to membrane transport, carbohydrate metabolism, amino acid metabolism. Nevertheless, *B*. *subtilis* DSM 32315 addition exerted little impact on the predicted pathways of cecal microbiota in broilers based on COG or KEGG database.

In conclusion, supplemental *B*. *subtilis* DSM 32315 improved the growth performance and intestinal structure of broilers, the possible mechanism for which was its role in shaping gut microbial composition. Specifically, *B*. *subtilis* DSM 32315 supplementation modified cecal microbiota of broilers towards a healthy balance by increasing the beneficial bacteria and decreasing the potentially harmful bacteria, as well as improving the interaction pattern within the community. This study can expand our fundamental knowledge concerning the roles of *B*. *subtilis* in the maintenance of intestinal microecological balance in animals.

## Materials and Methods

### Ethics Statement

All experimental protocols involving animals in this study were carried out in accordance with the institutional and national guidelines and approved by the Animal Care and Use Committee of Chinese Academy of Agricultural Sciences. Every effort was made to minimize animal pain, suffering, and distress.

### Animals and experimental design

A total of 240 1**-**day**-**old male Arbor Acre broiler chicks were randomly allocated into 2 groups with 10 replicates of each. Each replicate pen involving 12 birds. Initial body weights of birds were similar across all the replicates. Birds were received basal diets without or with 0.5 g/kg *B*. *subtilis* (DSM 32315, 2.0 × 10^9^ spores/g, Evonik Nutrition & Care GmbH, Germany) throughout the trial period. The basal diets were formulated based on the feeding standards of China (China′s Ministry of Agriculture, 2004) for broilers. The composition and nutrient levels are shown in Supplementary Table S1. All birds were raised in battery cages in an environmentally controlled room with continuous incandescent white light throughout the experiment. The room temperature was maintained at 33 °C for the first week, and then reduced by 3 °C per week until it reached 24 °C. Feed and fresh water were available *ad libitum*.

### Sample collection

At 42 d of age, birds were randomly selected from each replicate pen and slaughtered rapidly for sample collection. Intestinal segments (duodenum, jejunum, ileum and cecum) from these broilers (10 birds per group) were excised and weighed to determine the relative weight and length of them, which were expressed as the ratio of intestinal weight (g) and intestinal length (cm) to body weight (kg), respectively. The mid regions of duodenum, jejunum and ileum of these broilers (10 birds per group) were then cut off and fixed in 4% paraformaldehyde solution for morphology measurement. In addition, a little patch of ileum from each bird (10 birds per group) was harvested and rinsed, which was then quick**-**frozen in liquid nitrogen and kept at −80 °C for the quantification of gene expression. Besides, cecal content of broilers (7 birds per group) was collected and quick**-**frozen using liquid nitrogen, followed by storage at −80 °C for DNA extraction.

### Performance measurement

Body weight and feed intake were recorded for each replicate at 28 and 42 d of age. Average body weight at 28 and 42 d of age, along with average daily gain, average daily feed intake, and feed conversion ratio during the grower period (1−28 d) and overall period (1−42 d) were calculated.

### Intestinal morphological analysis

The cross-sections of fixed duodenal, jejunal and ileal tissues were obtained after staining with toluidine blue using standard paraffin-embedding procedures. For each sample, ten intact and straight villi in the section were chosen for morphology examination using a light microscope. Villus height was determined from the tip of villus to the junction of villus and crypt, while crypt depth was defined as the depth of invagination between adjacent villi, villus height to crypt depth ratio was calculated. For the purpose of statistical analysis, the average of these measured values was used.

### Quantitative real-time PCR for ileal genes

Total RNA was extracted from the ileum using Trizol Reagent (Invitrogen, Carlsbad, USA). Extracted RNA was dissolved in RNase**-**free water and quantified using an UV/Visible spectrophotometer (Amersham Bioscience, Sweden) at an absorbance of 260 nm. The quality of RNA was estimated from the absorbance ratio at 260 to 280 nm and by determination of the 18S and 28S bands after electrophoresis in 1% agarose gels stained with ethidium bromide. The relative mRNA expression of immune-related genes was determined using quantitative real-time PCR according to the procedures of Zhang *et al*.^54^. Gene specific primer sequences are shown in Supplementary Table S2. The results of relative mRNA expression of intestinal genes were calculated using the 2^−ΔΔCt^ method^55^.

### 16S rDNA sequencing of cecal microbiota

Total bacteria DNA was extracted from 200 mg of digesta from cecum using the E.Z.N.A.^TM^ Mag-Bind DNA Kit (Omega Bio-Tek, Norcross, USA) following the manufacturer′s instructions. The quality and concentration of extracted DNA were measured using gel electrophoresis and Qubit 2.0 fluorometer (Life Technology, Carlsbad, USA). Bacterial 16S rDNA sequences spanning the variable regions V3–V4 were amplified using primer 341 F (5′-CCCTACACGACGCTCTTCCGATCTG-3′) and 805 R (5′-GACTGGAGTTCCTTGGCACCCGAGAATTCCA-3′)^56^. The PCR mixture contained 15 μl of Taq Master Mix (Vazyme, Nanjing, China), 1 μL of forward and reverse primers, and 10 ng template DNA in a total volume of 30 μL. The amplification profile consisted of the following procedure: initial denaturation at 94 °C for 3 min, 5 cycles of three steps (94 °C for 30 s, 45 °C for 20 s, and 65 °C for 30 s), 20 cycles of three steps (94 °C for 20 s, 55 °C for 20 s, and 72 °C for 30 s), followed by a final step at 72 °C for 5 min. The PCR products were detected by agarose gel electrophoresis and purified using a PCR Purification kit (SAGON SK8131 kit) (Sangon, Shanghai, China) to remove excess primer dimers and dNTPs. 16S rDNA sequencing was carried out on the Illumina MiSeq 2 × 250 platform (Illumina, San Diego, USA) at Sangon Bioengineering Co. Ltd. (Shanghai, China). Raw tags were produced by merging paired-end reads using FLASH software (v1.2.7)^57^ and were demultiplexed and quality-filtered using QIIME (v1.7.0)^58^ to obtain the high quality clean tags. Besides, the chimera sequences were removed to obtain effective tags by using the UCHIME algorithm^59^. Clustering of filtered sequences into OTUs was achieved using UPARSE (v7.0.1001)^60^ at 97% sequence identity. Taxonomic classification at different taxonomic levels of OTU sequences were performed by comparing sequences to the GreenGene database^61^. Shannon and Simpson diversity indices, Chao1 and ACE richness estimators, as well as diversity coverage were included in α-diversity analysis by using the MOTHUR program^62^. The PCA and PCoA plots were used to estimate pairwise distances between samples and to establish β-diversity. In addition, LEfSe was employed to identify the biological differences between groups (*P* value less than 0.05 and LDA score higher than 2.0 were considered as significant). Correlation network analysis was used to uncover the internal interaction within the microbial community. Metagenome functional content from 16S rDNA was predicted using PICRUSt^63^ based on KEGG and COG databases. The results of pathway analysis of each sample were visualized in a heatmap generated by using R package (http://www.r-project.org).

### Statistical analysis

Data were presented as mean ± standard deviation. Pens were used as the experimental unit for growth performance parameters, whereas an individual bird served as the experimental unit for other parameters. The t-test (SPSS 18.0 software) was used to measure the effects of dietary treatment. Significance was defined as *P* < 0.05.

## Electronic supplementary material

Supplementary figures and tables
