# Supplementary material for: Supplemental Bacillus subtilis DSM 32315 manipulates intestinal structure and microbial composition in broiler chickens
Source: Sci Rep. 2018 Oct 18;8:15358. doi: 10.1038/s41598-018-33762-8 (PMC6194052; doi:10.1038/s41598-018-33762-8)
Supplement: Supplementary file 1 — Supplementary figures and tables [file 41598_2018_33762_MOESM1_ESM.docx]

# Supplemental *Bacillus subtilis* DSM 32315 manipulates intestinal structure and microbial composition in broiler chickens

Youbiao Ma^1,*^, Weiwei Wang^1,*^, Haijun Zhang^1^, Jing Wang^1^, Wenming Zhang^2^, Jun Gao^2^, Shugeng Wu^1,†^, Guanghai Qi^1,†^

*^1^Key Laboratory of Feed Biotechnology of Ministry of Agriculture, Feed Research Institute, Chinese Academy of Agricultural Sciences, Beijing 100081, China*

*^2^ Evonik Degussa (China) Co. Ltd., Beijing 100026, China*

^*^Youbiao Ma and Weiwei Wang contributed equally to this work. ^†^Correspondence and requests for materials should be addressed to S.G. Wu (email: [wushugeng@caas.cn](mailto:wushugeng@caas.cn)) or G.H. Qi (email: qiguanghai@caas.cn).

| Ingredients | Stage | | |
| --- | --- | --- | --- |
|  | 1−14 d | 15−28 d | 28−42 d |
| Corn | 582.0 | 587.0 | 610.0 |
| Soybean meal | 348.6 | 330.8 | 308.5 |
| Soybean oil | 25.4 | 44.8 | 47.2 |
| Dicalcium phosphate | 22.7 | 20.0 | 18.2 |
| Limestone | 8.2 | 7.2 | 7.0 |
| Sodium chloride | 3.5 | 3.5 | 3.5 |
| DL**-**Methionine (98%) | 3.4 | 2.6 | 2.0 |
| L**-**lysine.HCl (99%) | 3.1 | 1.5 | 0.4 |
| Multivitamin^1^ | 0.2 | 0.2 | 0.2 |
| Multimineral^2^ | 2.0 | 2.0 | 2.0 |
| Choline chloride (50%) | 1.0 | 1.0 | 1.0 |
| Nutrient levels |  |  |  |
| Metabolic energy (MJ/kg) | 12.35 | 129.7 | 131.8 |
| Crude protein | 220.0 | 210.0 | 200.0 |
| Calcium | 10.0 | 10.0 | 9.0 |
| Available phosphorus | 5.0 | 4.5 | 4.0 |
| Lysine | 12.7 | 11.0 | 10.5 |
| Methionine | 5.5 | 5.2 | 4.8 |

**Supplementary Table S1.** Composition and nutrient levels of diets (g/kg). ^a^ Supplied per kg of diet: vitamin A, 12 500 IU; vitamin D3, 2500 IU; vitamin K3, 2.65 mg; vitamin B1, 2 mg; vitamin B2, 6 mg; vitamin B12, 0.025 mg; vitamin E, 30 IU; biotin, 0.0325 mg; folic acid, 1.25 mg; pantothenic acid, 12 mg; niacin, 50 mg. ^b^ Supplied per kg of diet: Cu, 8 mg; Zn, 75 mg; Fe, 80 mg; Mn, 100 mg; I, 0.35 mg, Se, 0.15 mg.

| Genes | Primer sequence (5′-3′) | Accession no. |
| --- | --- | --- |
| β-actin | F: ATCCGGACCCTCCATTGTC | NM_205518 |
|  | R: AGCCATGCCAATCTCGTCTT |  |
| TLR-4 | F: CCATCCACTCAGACAACCTTTCCA | AY064697 |
|  | R: AGTAAACGCAGCAGACGCG |  |
| TLR-2 | F:ACATGTGTGAATGGCCTGAA | NM_204278 |
|  | R:TTGAGAAATGGCAGTTGCAG |  |
| IL-4 | F: GTGCCCACGCTGTGCTTAC | AJ621249 |
|  | R: AGGAAACCTCTCCCTGGATGTC |  |
| IL-1β | F: ACTGGGCATCAAGGGCTACA | NM 204524 |
|  | R: GCTGTCCAGGCGGTAGAAGA |  |
| TNF-α | F: TACTCAGGACAGCCTATGCCAACAA | AY765397.1 |
|  | R: GGAAGGGCAACTCATCTGAACTGG |  |
| IL-10 | F: GACCAGCACCAGTCATCAGCA | NM_001004414.2 |
|  | R: GCTTTGTAGATCCCGTTCTCATCC |  |
| IL-6 | F: AAATCCCTCCTCGCCAATCT | NM_204628.1 |
|  | R: CCCTCACGGTCTTCTCCATAAA |  |
| IFN-γ | F: GACAAGTCAAAGCCGCACAT | NM_205149.1 |
|  | R: CAAGTCGTTCATCGGGAGC |  |

**Supplementary Table S2.** Primers used for quantitative real-time PCR. TLR, toll-like receptor; IL, interleukin; TNF, tumor necrosis factor; IFN, interferon.

| 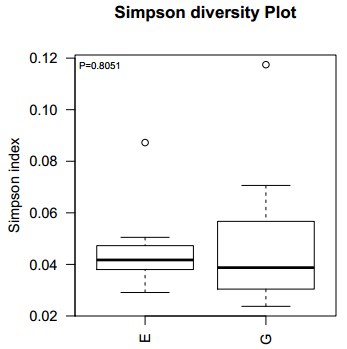 | 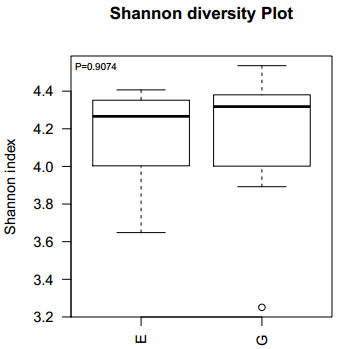 |
| --- | --- |
| 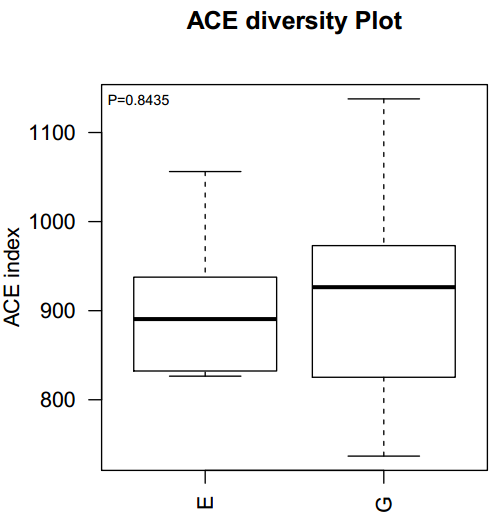 | 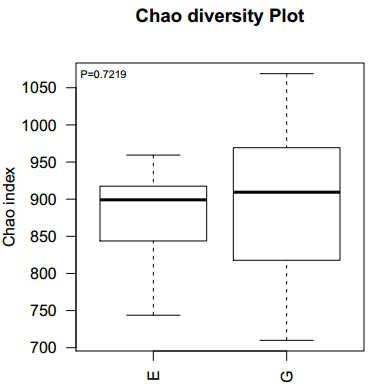 |
| 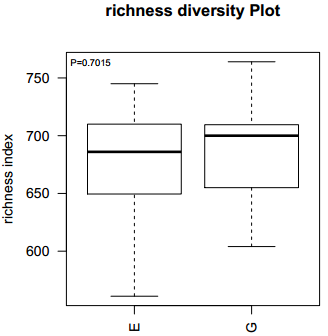 |  |

**Supplementary Figure S1.** Alpha diversity of cecal microbial sequencing. E, control group; G, treatment group. E, control group; G, treatment group.

| A. At order taxonomic level |
| --- |
| 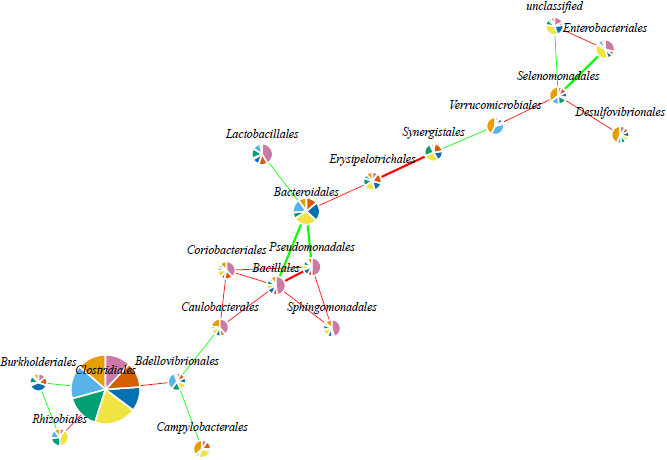  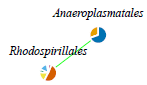  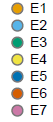 |
| 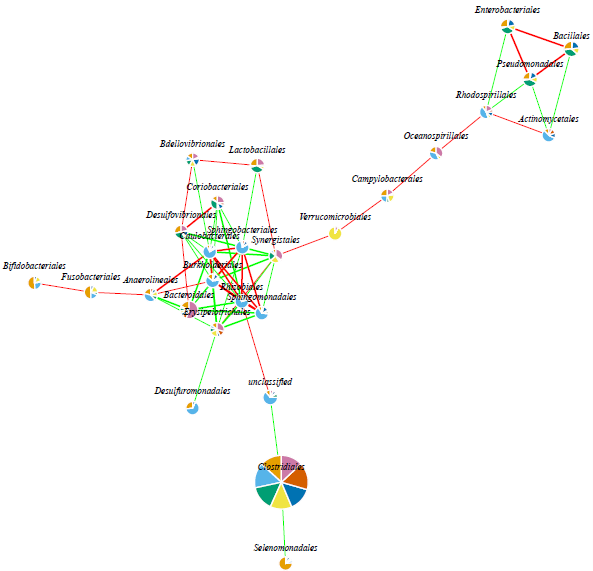  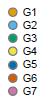  B. At family taxonomic level |
| 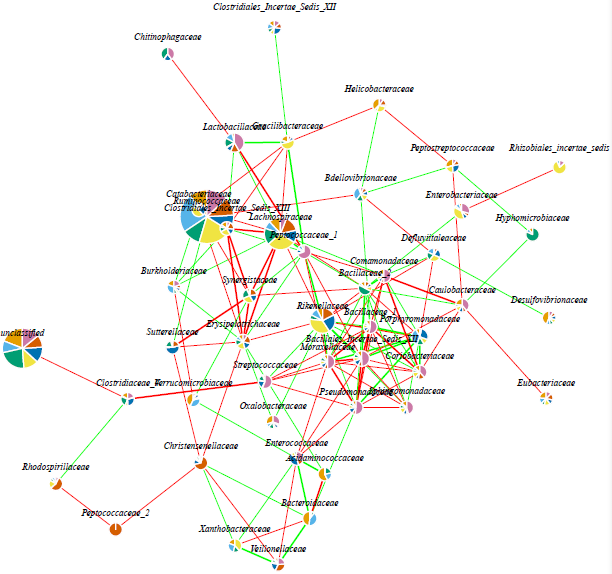  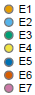 |
| 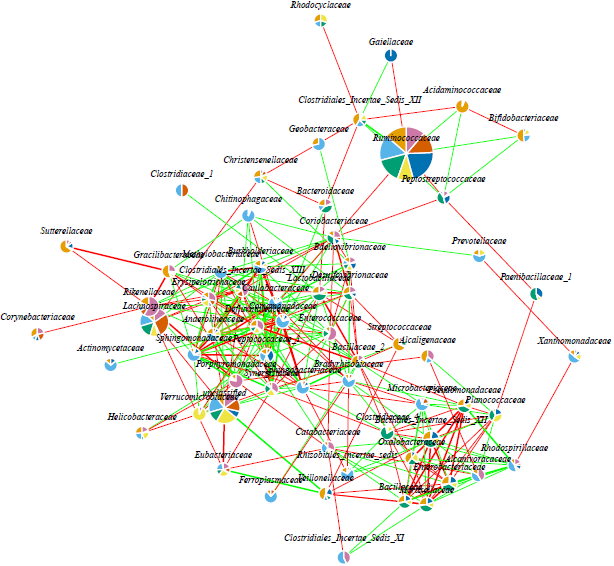  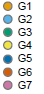 |

**Supplementary Figure S2.** The co-network pattern of cecal microbiota at order (A) and family (B) levels in broiler chickens (*n* = 7). The significance of correlation was set as *P* < 0.05 and expressed by the dotted line, while the high significance of correlation was set as *P* < 0.01 and expressed by the full line. The red and green lines among nodes stand for the positive and negative correlations, respectively. The weight of the lines correspond to the correlation coefficients, whose values were higher and lower than 0.8 were represented by the thick and thin lines, respectively. The diameter of the nodes was proportional to the relative abundance. Taxa in brackets are based on annotations suggested by the Greengenes database. E, control group; G, treatment group.

| 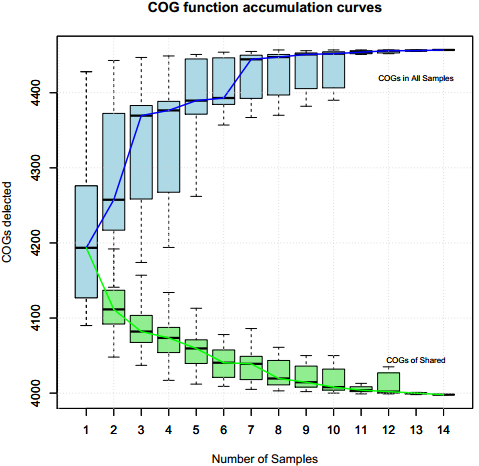 | 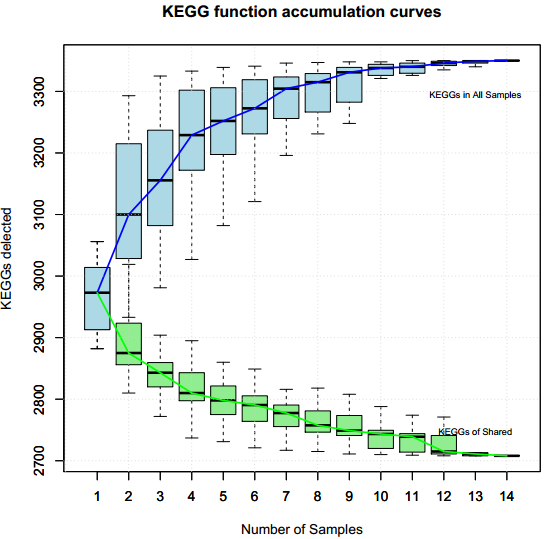 |
| --- | --- |

**Supplementary Figure S3.** Functional accumulation curves based on Clusters of Orthologous Groups (COG) and Kyoto Encyclopedia of Genes and Genome (KEGG) databases. The ordinate represents the number of COG ID, which corresponded to the sample amount represented by the abscissa. The blue and green bars signify the failarmy and intersection of the total COG ID from all samples, respectively.


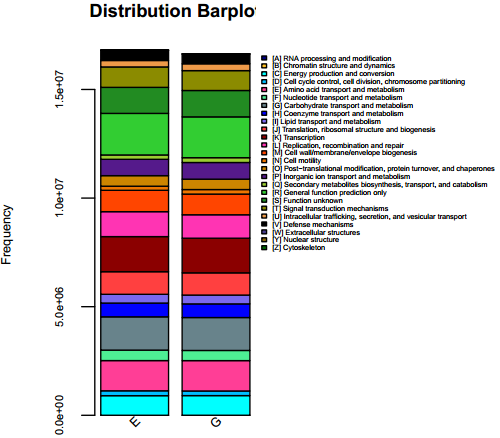


**Supplementary Figure S4.** The composition of functional genes of cecal microbiota based on Clusters of Orthologous Groups (COG) in broiler chickens. E, control group; G, treatment group.


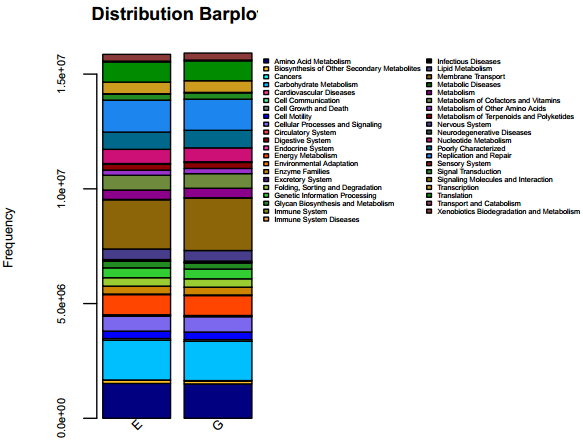


**Supplementary Figure S5.** The composition of functional genes of cecal microbiota based on Kyoto Encyclopedia of Genes and Genome (KEGG) in broiler chickens. E, control group; G, treatment group.

| **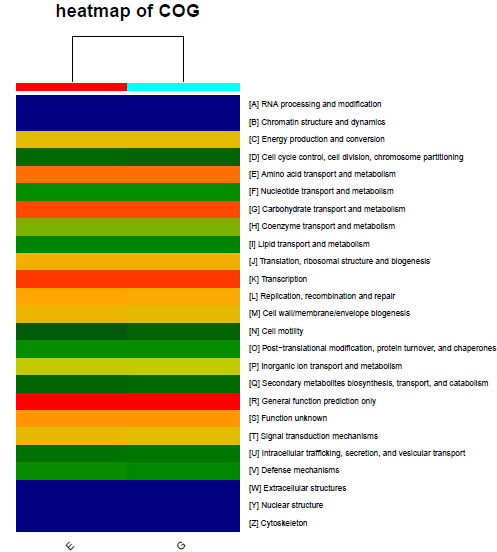** | |
| --- | --- |
| **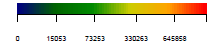** |  |

**Supplementary Figure S6.** Heatmap of functional pathways of cecal microbiota based on Clusters of Orthologous Groups (COG) in control and treatment groups. Colors reflect relative abundance from low (green) to high (red). E, control group; G, treatment group.

| 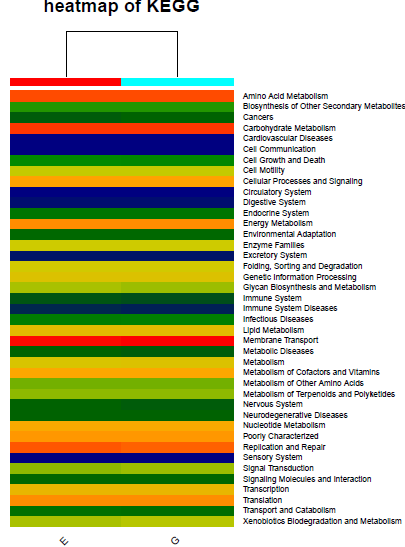 | |
| --- | --- |
| 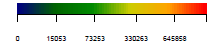 |  |

**Supplementary Figure S7.** Heatmap of functional pathways of cecal microbiota based on Kyoto Encyclopedia of Genes and Genome (KEGG) in control and treatment groups. Colors reflect relative abundance from low (green) to high (red). E, control group; G, treatment group.

|  |  |
| --- | --- |
